# Induction of Osteogenic Differentiation of Mesenchymal Stem Cells by Bioceramic Root Repair Material

**DOI:** 10.3390/ma12142311

**Published:** 2019-07-19

**Authors:** Hadeel Y. Edrees, Sawsan T.H. Abu Zeid, Hazem M. Atta, Mehal A. AlQriqri

**Affiliations:** 1 Endodontic Department, Faculty of Dentistry, King Abdulaziz University, Jeddah 22252, Saudi Arabia; 2 Endodontic Department, Faculty of Oral and Dental Medicine, Cairo University, Cairo 12345, Egypt; 3 Clinical Biochemistry Department, Faculty of Medicine-Rabigh, King Abdulaziz University, Jeddah 21589, Saudi Arabia; 4 Medical Biochemistry and Molecular Biology Department, Faculty of Medicine, Cairo University, Cairo 11562, Egypt; 5 Stem Cell Unit, King Fahad Medical Research Center, King Abdulaziz University, Jeddah 23839, Saudi Arabia

**Keywords:** mesenchymal stem cells, root repair materials, alkaline phosphatase, mineral trioxide aggregates, endosequence, calcium silicate cement

## Abstract

This study aimed to evaluate the osteogenic activity of Endosequence Root Repair Material (ERRM) putty using rat mesenchymal stem cells (MSCs). The extract of set ERRM and ProRoot-mineral trioxide aggregate (MTA) (control) was cocultured with rat MSCs and incubated for one, three, and seven days. The cell viability and proliferation were assessed. A quantitative real-time polymerase chain reaction for bone morphogenetic protein-2 (BMP-2), alkaline phosphatase, bone sialoprotein, and osteocalcin gene expression was performed. Both materials enhanced cell viability and proliferation, which increased over time. On day seven, the cells treated with either material exhibited significantly greater cell viability compared with control untreated cells. MSCs treated with either material showed deeper alkaline phosphatase staining after three days compared to control untreated cells. Treated MSCs also exhibited upregulation of the gene expression of bone morphogenetic protein-2, alkaline phosphatase, bone sialoprotein, and osteocalcin. Both ERRM and ProRoot-MTA enhance the osteogenic differentiation of MSCs.

## 1. Introduction

For several years, mineral trioxide aggregate (MTA) was used as the standard root repair material [1]. MTA is a calcium-silicate-based cement, and it is the material of choice to improve the communication between the dental pulp and periodontal tissues, with clinical as well as histological success [1,2]. Its main drawbacks are the prolonged setting time (several hours) and its difficult handling. Although the fresh mix of MTA was reported to be biocompatible due to its calcium hydroxide byproduct [3,4], its aged material, cured for 28 days, exhibited reduced cell growth [4]. Many studies reported certain cytotoxicity of MTA on macrophages and fibroblasts [5], and reduced cell growth 28 days after hydration [3].

Several manufacturers developed a new root repair material made from bioceramics. Endosequence Root Repair Material (ERRM) (Brasseler, Savannah, GA, USA) is available in premixed packages (putty) that do not require preparation before use. It is mainly composed of calcium silicate, monobasic calcium phosphate, calcium hydroxide, and zirconium oxide. It has antibacterial [6] and antifungal activities [7].

It is claimed that the material is biocompatible with human periodontal tissues [8]. ERRM was found to be significantly less injurious to tissues than MTA when implanted in rat subcutaneous tissues after seven and 30 days [9]. Additionally, ERRM enhanced an apical seal with increased tissue healing when used as a retrograde filling [10]. To date, there have been limited studies on ERRM cytotoxicity, and the existing studies have come to varying conclusions. Some studies found no difference between ERRM and MTA regarding cell viability [11,12]. However, other studies showed that ERRM had lower cell viability in some conditions [13,14]. One study found that ERRM elutes reduced the bioactivity and Alkaline phosphatase ALP activity of osteoblast-like cells [13]. Although Damas et al. 2011, when comparing MTA Angelus with ProRoot-MTA and ERRM, discovered that all the tested materials demonstrated cell viability >91%. However, the root repair material in putty form showed statistically significantly less cell viability when compared with the other materials [14].

The aim of the current study was to evaluate the cell viability and osteogenic activity of putty paste Endosequence Root Repair Material (ERRM) compared with white ProRoot-MTA (Dentsply Tulsa Dental Specialties, Tulsa, OK, USA) through the use of various tests such as a proliferation assay, alkaline phosphatase staining, and a reverse transcription polymerase chain reaction, and quantitative real-time polymerase chain reaction using rat mesenchymal stem cells (MSCs). The null hypothesis was that there was no difference between the investigated materials with regard to their cell viability and osteogenic behavior on MSCs.

## 2. Materials and Methods

The ethical approval (#057-15) for this work was obtained from the King Abdulaziz University Ethics Committee at the beginning of the current study. The institutional guidelines for animal care were adopted.

### 2.1. Material Extract Preparation

Under sterile conditions, three discs (10 mm diameter and 3 mm height) of both ERRM and ProRoot-MTA were used according to the manufacturers’ instructions. ERRM is available as a putty paste and was used without further preparation. A homogenous paste of ProRoot-MTA was prepared by mixing the powder with its liquid (in a 3:1 ratio). Each disc weighed approximately 24 mg. The discs were covered with sterile gauze moistened with sterile deionized water and placed in an incubator at 37 °C for 3 days to set [13], and then exposed to UV light for 1 h for sterilization.

The discs of each material were inserted into a culture tube containing 20 mL Dulbecco’s Modified Eagle Medium (DMEM; Gibco, Life Technology, Carlsbad, CA, USA) and 10% fetal bovine serum (FBS; Gibco, Life Technology, Carlsbad, CA, USA) to obtain extracts of the active agent. After 3 days, the culture media underwent sterile filtration to remove any grains of soluble material [15,16]. The pH of the extracts was determined using a pH meter (HANNA pH 211, UVP Inc., UpLand, Smithfield, RI, USA) and was 11 for the ERRM and 11.3 for the ProRoot-MTA.

### 2.2. Preparation of Rat Bone Marrow-Derived MSCs

Rat bone-marrow-derived MSCs provided by the King Fahad Centre for Medical Research at King Abdulaziz University were used. All animal procedures and caretaking were carried out in accordance with the institutional guidelines.

The procedure was performed in the Stem Cell Unit according to a previously published method [17]. MSCs were extracted and suspended in 25 cm^2^ cell culture flasks (Corning, Corning, NY, USA) in DMEM media containing 10% FBS and supplemented with antibiotics (100 U/mL penicillin/streptomycin (Gibco, Life Technology, Carlsbad, CA, USA)) [15]. The cells were incubated at 37 °C in an 85% humidified atmosphere containing 5% carbon dioxide. These cells were confirmed to be MSCs by flow cytometry (FACSAria III cell sorter model no. 6482-35, BD Bioscience, Erembodegem, Belgium) for detection of CD29^+^/CD45^−^ cells [17]. For the gene expression studies, stem cells were seeded at a density of 3 × 10^5^ cells/well in a 6-well cell culture plate. For the cell proliferation assay, MSCs were seeded at a density of 1 × 10^5^ cells/well in a 96-well cell culture plate containing DMEM, 2% FBS, and antibiotics supplemented with 10 mmol/L glycerophosphate [16]. They were incubated for 24 h before treatment with the extracts of the investigated materials.

The seeded cells were treated with the extract of each investigated material and incubated for 1, 3, and 7 days (6 wells for each incubation period) at 37 °C in an 85% humidified atmosphere containing 5% carbon dioxide [18]. For each incubation period, 6 wells of seeded cells were left untreated and served as controls.

### 2.3. Cell Viability and Proliferation Assay

After each incubation period, 10 µL of WST-1 Cell Proliferation Assay Reagent (WST-1 Cell Proliferation Reagent Kit, Sigma-Aldrich, Inc., Munich, Germany) was added for an additional 4 h. Finally, solvent (sodium dodecyl sulfate, SDS) was added and incubated for 10–15 min. Cell growth, viability, and proliferation were then determined by measuring the absorbance at 450 nm using an ELISA reader (ELx808, Biotek, Winooski, VT, USA). To ensure the accuracy of the results, the procedure was repeated three times. The treated and untreated culture plates, at different incubation periods, were also examined under a scanning electron microscope (FE SEM, 450 FEI, Amsterdam, The Netherlands). 

### 2.4. Alkaline Phosphatase Staining

After each incubation period, the cultured cells were fixed with 70% ethanol and then washed 3 times with deionized water. In each well, 300 µL of alkaline phosphatase live stain solution (1-Step NBT/BCIP solution; Thermo Fisher Scientific, Inc., Rockford, IL, USA) was applied for 15 min. The stained cells were then evaluated for alkaline phosphatase production under light microscopy and photographed. 

### 2.5. Reverse-Transcription Polymerase Chain Reaction (RT-PCR) and Quantitative Real-Time Polymerase Chain Reaction (qPCR)

After each incubation period, MCSs were subjected to a quantitative real-time polymerase chain reaction (qPCR) and microarray-based comparative genomic hybridization using the StepOnePlus^TM^ Real-Time PCR system (AB Applied Biosystem, Life Technology, Foster, CA, USA). Reverse transcription of RNA to complementary deoxyribonucleic acid (cDNA) was performed using a cDNA reverse transcription kit (ImProm-II™ Reverse Transcription System, Madison, WI, USA). The reaction was performed using a thermal cycler at 25 °C for 5 min, 42 °C for 120 min, and 70 °C for 15 min. The qPCR was then performed using the QuantiFast^®^ SYBR^®^ Green PCR Kit (master cat no. 204054; (QIAGEN GmbH, QIAGEN Strasse, Hilden, Germany) with initial denaturation at 95 °C for 10 min, followed by 34 cycles at 95 °C for 5 s, 65 °C for 10 s, and 72 °C for 15 s. The quantitative analysis of the expression of genes related to osteogenic differentiation was performed using the following rat-specific primers: bone morphogenetic protein-2 (rBMP-2 forward: 5′-AACACCGTGCTCAGCTTCCA-3′, reverse: 5′-TTCCCACTCATTTCTGAAAGT-3′), osteocalcin (rOC-2 forward: 5′-TACCAGGGAGGTGTGTGA-3′ reverse: 5′-CATAGATGCGCTTGTAGG-3′), alkaline phosphatase (rAlp-1 forward: 5′-GCTCATTTCCAACATCATGGTC-3′, reverse: 5′-ACTGGTCAGAGTCACCTG-3′), bone sialoprotein (rBsp-1 forward: 5′-GATAGTTCGGAGGAGGAG-3′, reverse: 5′-TTACCCCTGAGAGTATGG-3′), and GAPDH (rGapdh-2 forward: 5′-AGTCCATGCCATCATTGC-3′, reverse: 5′-GCAGGGATGATGTTCTGG-3′) as the endogenous control gene [19].

The mRNA relative expression was quantitatively determined using the comparative cycle threshold (CT) method to calculate (2^−ΔΔCT^) [20]. First, (ΔCT) was calculated as the difference between the CT mean of each target gene and that of the endogenous control gene (GAPDH). Then, ΔΔCT was calculated as the differences of the ΔCT values of the calibrator samples and the ΔCT values of the test samples. Finally, the fold change, or the mRNA expression level, was calculated (2^−ΔΔCT^).

### 2.6. Statistical Analysis

ANOVA and Tukey’s post hoc statistical analyses were applied to the cell growth data using SPSS Windows software (Version, 16, Munich, Germany) at a significance level of 0.05.

## 3. Results

### 3.1. Cell Viability and Proliferation

**Figure 1:**
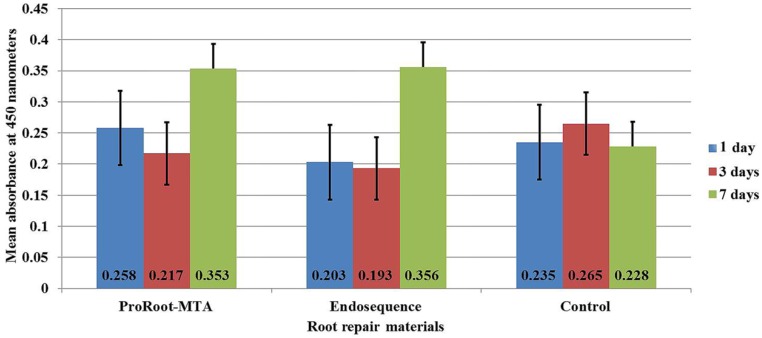
Error bar of means of mesenchymal stem cell viability with and without treatment with root repair material extracts at the different incubation periods.

The viability of cultured MSCs varied within the experimental periods according to the effect of the material used (Figure 1). There was only a significant difference between the groups on day seven (F = 8.534, *p* = 0.003). At day one, the treatment with ProRoot-MTA showed the greatest cell viability (mean 0.258 ± 0.05), whereas the ERRM putty showed the least viability (mean 0.203 ± 0.064) when compared to the control group (mean 0.235 ± 0.1). At day three, both root repair materials exhibited lower values of cell viability for ProRoot-MTA (mean 0.217 ± 0.052) and for ERRM putty (0.193 ± 0.073) than the control group (mean 0.265 ± 0.102); however, this difference was not statistically significant (*p* = 0.296).

After seven days, the viability of MSCs significantly increased after treatment with either of the root repair materials, ERRM putty or ProRoot-MTA (mean values 0.356 ± 0.073 and 0.353 ± 0.041, respectively) compared to the control group (0.228 ± 0.065) (Figure 1).

### 3.2. Scanning Electron Microscopy

**Figure 2:**
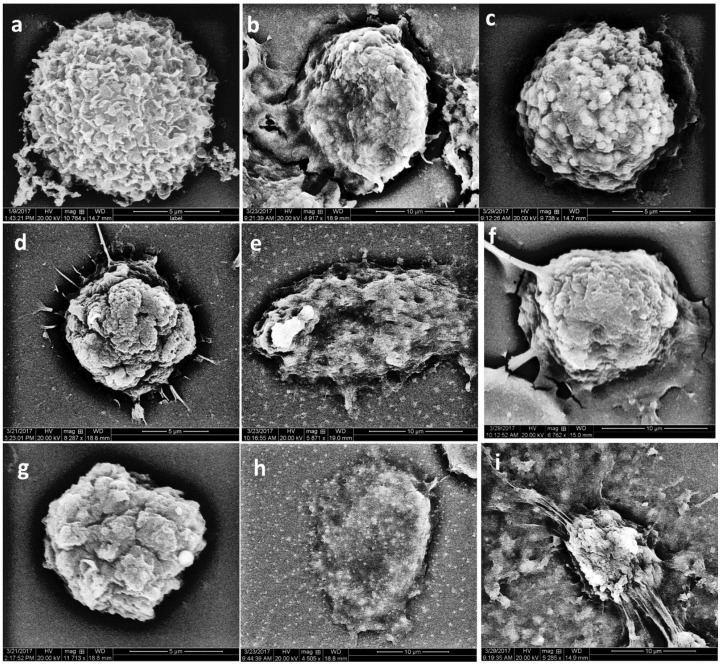
Scanning electron photomicrographs of control untreated mesenchymal stem cells (**a**–**c**), and those treated with Endosequence Root Repair Material (ERRM) (**d**–**f**) or ProRoot-mineral trioxide aggregate (MTA) extract (**g**–**i**) incubated for one, three, and seven days, respectively.

The control untreated MSCs appeared as a clump of spherical-shaped undifferentiated cells on day one (Figure 2a). On day three, they showed some elongation in their shape (Figure 2b), with some apoptotic changes (Figure 2c). 

On day one, after treatment with ERRM extract, the cells showed no relative changes in their shape. Some of the cells showed differentiation to osteoblast-like cells that possessed active processes (Figure 2d). On day three, the cell elongation became more prominent (Figure 2e). Some of the cells became spindle- or fusiform-shaped with evidence of cell division (cell mitosis). On day seven, the mitotic activity became more prominent, with further differentiation to osteoblast- or fibroblast-like cells (Figure 2f).

The cells treated with ProRoot-MTA extract appeared as undifferentiated cells with no changes compared to the control untreated cells (Figure 2g). On day three, the cells differentiated into osteoblast-like cells (Figure 2h). On day seven, the cells showed further differentiation with an active process (Figure 2i).

### 3.3. Alkaline Phosphatase Staining Analysis

**Figure 3:**
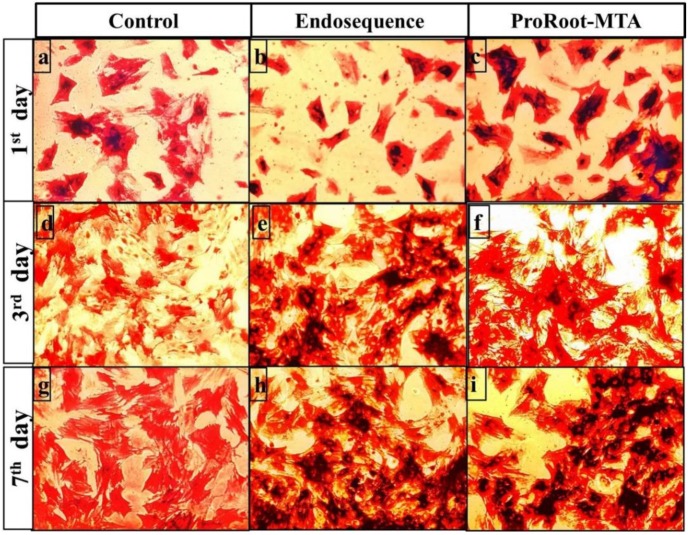
Photomicrographs of mesenchymal stem cells untreated and treated with either ERRM or ProRoot-MTA, stained with alkaline phosphatase staining on day one (**a**–**c**), day three (**d**–**f**) and day seven (**g**–**i**), respectively, at 20× magnification.

Figure 3 shows the light microphotographs of alkaline phosphatase staining of control untreated MSCs and of those treated either with ERRM or ProRoot-MTA root repair material after incubation for one, three, and seven days.

On day 1, there was no obvious staining difference between control untreated MSCs and those treated with either of the investigated extracts (Figure 3a–c). On day three, the ERRM-treated cells (Figure 3e) showed deeper staining than the ProRoot-MTA-treated cells (Figure 3f), whereas the control untreated cells showed lighter staining (Figure 3d). On day seven, both ERRM- and ProRoot-MTA-treated MSCs, which were not substantially different from each other, showed deeper staining than the control untreated cells (Figure 3g–i).

### 3.4. Reverse-Transcription Polymerase Chain Reaction and Quantitative Real-Time Polymerase Chain Reaction

**Figure 4:**
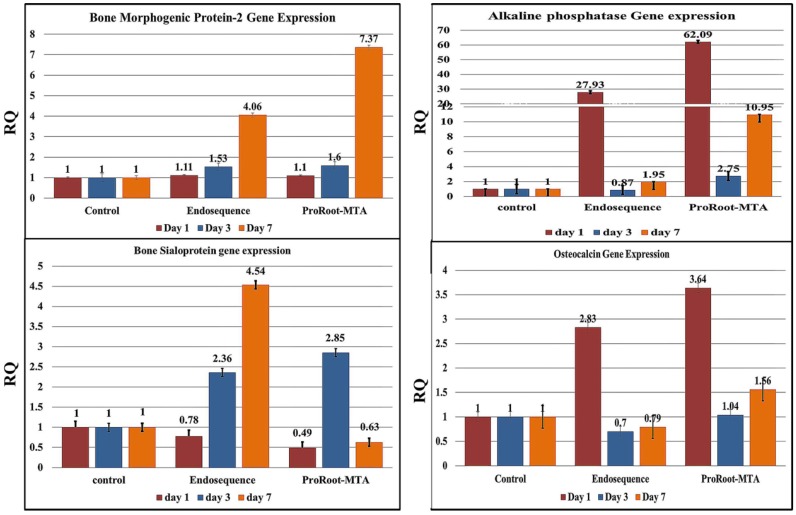
Relative quantification graphs of the ΔΔCT values of the mRNA expression level of target genes, including BMP-2, alkaline phosphatase, bone sialoprotein, and osteocalcin, after treatment with material extracts compared to control untreated mesenchymal stem cells at *p* < 0.05. The relative quantification of control cells was normalized to 1. The relative quantification (RQ) values of treated cells that are greater than 1 indicate upregulation, and values less than 1 indicate downregulation.

The mean CT and standard deviation were calculated for the reference (GAPDH) and target genes, including BMP-2, alkaline phosphatase, bone sialoprotein, and osteocalcin genes as markers for osteogenic activity, after cultured MSCs were exposed to root repair material extracts (Figure 4).

#### 3.4.1. Bone Morphogenetic Protein-2 (BMP-2) 

Compared with control untreated MSCs, the cultured cells exposed to both ERRM and ProRoot-MTA extracts exhibited significant upregulation of BMP-2 gene expression, which increased over time (Figure 4). The ProRoot-MTA extract induced significant expression on the seventh day (7.37).

#### 3.4.2. Alkaline Phosphatase

The ProRoot-MTA extract induced a significantly greater mean CT value of gene expression (62.09) on day one than the ERRM extract (27.93 at *p* < 0.05). This expression markedly decreased on day three and was then re-upregulated on day seven (Figure 4).

#### 3.4.3. Bone Sialoprotein

Both extracts induced downregulation of gene expression on day one. On day three, ProRoot-MTA induced greater upregulation (2.85), then down-regulation (0.63) on day seven. The ERRM extract induced upregulation on day three (2.36) that gradually increased until day seven (4.54) (Figure 4).

#### 3.4.4. Osteocalcin

Both extracts exhibited upregulation of osteocalcin gene expression on day one with a greater value obtained by ProRoot-MTA treatment (3.64) than by ERRM treatment (2.83). These values were markedly decreased on day three and then re-upregulated on day seven (Figure 4).

## 4. Discussion

MSCs have the capacity to undergo ex vivo differentiation into several cell lineages (such as osteoblast-, cementoblast-, and/or odontoblast-like cells) upon favorable environmental conditions. This enables their use for in vitro biocompatibility studies of new dental materials [21,22]. In the current study, rat bone-marrow-derived MSCs were used to evaluate the osteogenic activity of ERRM putty in comparison with the conventional white MTA (ProRoot-MTA). Because the direct contact of the repair material with MSCs may inhibit cell growth [3], and because its tissue reaction results in leaching into the surrounding environment [23,24], material extracts were used in the current study [14].

The justification for using one concentration of extract material in this study was based on previous evaluations by Lee et al. and Braga et al. in 2014 of the effect of different concentrations of the extract of MTA on the viability of stem cells. They found that there was no significant difference in the cell viability between 1:1, 1:2, 1:4, and 1:8 concentrations [15,16]. Thus, one concentration was chosen for this study.

Upon SEM examination, there was no obvious difference in the shape of control MSCs during the seven-day experimental period. Both investigated extracts produced significant increases in the viability of MSCs up to day seven. Increases in the rate of cell proliferation of dental pulp cells were previously determined for both ProRoot-MTA and ERRM up to day four, which then decreased on day seven compared to control untreated cells [25]. On day one, the cells treated with either of the extracts showed differentiation into osteoblast-like cells with no statistically significant difference. On day three, there was evidence of mitotic cell division, with further advances on day seven. The acceptable level of MSC viability after using either of the material extracts may be attributed to the calcium silicate composition of the extracts [26].

To elucidate the mechanism of osteogenic induction by the investigated materials, related gene expression (such as BMP-2, alkaline phosphatase, bone sialoprotein, and osteocalcin) was evaluated by qPCR. The bone formation and bone remodeling processes take place in various stages and are associated with different specific markers in each stage of osteoblast differentiation and mineralization. BMP-2 is an essential transforming growth factor expressed in the early stages of MSC differentiation into calcified tissue-forming cells (such as osteoblast- or odontoblast-like cells) [27,28]. It has been shown that MTA-induced expression of BMP-2 after 5 h of incubation on rat dental pulp cells [29] is associated with increases of extracellular calcium ion concentration [21,29]. The present findings support the importance of BMP-2 gene expression in the induction of osteogenesis. The extracts of both tested materials induced its expression, starting from day one of incubation, with gradual increases up to day seven in comparison to the control. This finding was in agreement with the proliferation and full differentiation of MSCs into osteoblasts as determined by SEM.

Once the undifferentiated mesenchymal cells become well-differentiated osteoblasts, the osteoblasts induce the expression of Runt-related transcription factors that regulate the genes related to osteogenesis, including alkaline phosphatase, bone sialoprotein, and osteocalcin [30].

The present results showed significant expression of alkaline phosphatase genes by stem cells exposed to ProRoot-MTA extracts for one day compared to those exposed to ERRM extract. This finding may indicate the greater osteogenic induction potential of ProRoot-MTA compared to ERRM putty repair material. This expression gradually decreased over time up to seven days. Alkaline phosphatase is an essential early differentiation marker released by osteoblasts and/or odontoblasts in the initial stage of calcified tissue formation [25,31,32], during the initiation of mineralization to allow the formation of hydroxyapatite [31,32]. It was reported that alkaline phosphatase exhibited its maximum expression by differentiated osteoblasts within the first few days and then gradually declined on days seven and 14 [33]. The present findings showed that alkaline phosphatase levels were maintained at higher levels up to the end of the experiment (day seven), which was sufficient to promote further cell differentiation and osteogenic gene expression [34].

Both of the repair materials investigated are calcium silicate-based materials. Calcium hydroxide is a byproduct of their hydration reaction [4]. The dissociation of calcium hydroxide to calcium ions and hydroxyl ions plays an essential role in the osteogenic activity of stem cells. Furthermore, a pH over 8 [23] can activate osteoblasts to express the alkaline phosphatase activity [35], which is considered an important indicator for the formation of active calcified tissue [33]. Not only calcium but also silicate ions are released from the calcium silicate material into the surrounding environment [23], upregulating gene expression related to cell differentiation and osteogenic activity [21]. The pH and calcium release of ERRM and MTA extracts was reported in previous studies [23,24]. The ERRM exhibited high alkaline pH ~11 from day one up to the seventh day. A high amount of calcium ions was released (100.26 mg/L) on the first day, which gradually decreased over time to 75.7 mg/L on day seven. Regarding MTA, pH measured 11.5 from day one up to the seventh day. It exhibited high calcium ion release from day one 64.3 mg/L [24].

Regarding the released phosphorus, aluminum and silicon, ERRM leached out around 1.6, 14.4, and 0.46 mg/L respectively, whereas MTA leached-out around 0.19, 0.16, and 0.07 mg/L respectively [24]. It was believed that the released silicon and phosphorus, as well as calcium ions, had strong effects on dentin mineralization and new bone growth [36,37].

Bone sialoprotein and osteocalcin are major noncollagenous proteins expressed by fully differentiated osteoblasts and/or odontoblasts in relation to late-stage calcified tissue formation, and the regulation of the formation and growth of hydroxyapatite crystal of bone and cementum mineralization [31,35,38]. Bone sialoprotein is a marker that indicates an increased mineralization level of osteoblasts [39]. Osteocalcin is the late osteoblast differentiation marker, which has increased expression over time from day seven up to day 21 [31]. This finding may be attributed to the role of osteocalcin for binding of hydroxyapatite crystals in the extracellular calcified matrix [31]. This finding was supported by Zhao and co-authors [40], who proved the upregulation of alkaline phosphatase, bone sialoprotein, and osteocalcin gene expression by MTA.

It has been suggested that bone sialoprotein is expressed in the middle stage of osteoblast differentiation, earlier than osteocalcin, which is expressed as a late-stage marker [41]. In the current study, due to the therapeutic effect of both ERRM and ProRoot-MTA extract, osteocalcin was highly expressed on day one, whereas bone sialoprotein gradually increased over time. Bone sialoprotein expression was induced more by ERRM, while osteocalcin induction was greater with ProRoot-MTA. This finding was in agreement with a study that reported that white MTA extract induced the expression of BMP-2, osteopontin, and osteocalcin at higher levels compared to iRoot extract [25]. Thus, both ERRM and MTA promoted cell proliferation and differentiation, with MTA promoting notable gene expression. One of the limitations of this study is the use of rat MSC instead of human stem cells, which would have been more meaningful, due to availability.

The choice of the material to be used clinically not only depends on the bioactivity of the material but also on different factors such as the setting time, discoloration possibility, handling properties, and cost. MTA has the disadvantages of having a long setting time, being difficult to handle, and having discoloration potential [2]. ERRM is clinically easier to manipulate [42] and has a lesser risk of tooth discoloration [43]. Therefore, further studies should be done to evaluate the clinical outcome of both materials.

## 5. Conclusions

The results of the present study indicate that ERRM and ProRoot-MTA are biocompatible materials, as proven by the enhanced differentiation of MSCs into osteoblast-like cells with notable gene expression related to MTA. Both materials can induce osteogenic differentiation of MSCs as they upregulated the gene expression of BMP-2, alkaline phosphatase, bone sialoprotein, and osteocalcin, which are responsible for cell differentiation, maturation, and calcification.
